# Trends in surface equivalent potential temperature: A more comprehensive metric for global warming and weather extremes

**DOI:** 10.1073/pnas.2117832119

**Published:** 2022-01-31

**Authors:** Fengfei Song, Guang J. Zhang, V. Ramanathan, L. Ruby Leung

**Affiliations:** ^a^Frontier Science Center for Deep Ocean Multispheres and Earth System and Physical Oceanography Laboratory, Ocean University of China, Qingdao 266005, China;; ^b^Qingdao National Laboratory for Marine Science and Technology (QNLM), Qingdao 266005, China;; ^c^Atmospheric Sciences and Global Change Division, Pacific Northwest National Laboratory, Richland, WA 99354;; ^d^Scripps Institution of Oceanography, University of California San Diego, La Jolla, CA 92093

**Keywords:** global warming, surface equivalent potential temperature, atmospheric convection, weather extremes

## Abstract

Trends in surface air temperature (SAT) are a common metric for global warming. Using observations and observationally driven models, we show that a more comprehensive metric for global warming and weather extremes is the trend in surface equivalent potential temperature (Thetae_sfc) since it also accounts for the increase in atmospheric humidity and latent energy. From 1980 to 2019, while SAT increased by 0.79°C, Thetae_sfc increased by 1.48°C globally and as much as 4°C in the tropics. The increase in water vapor is responsible for the factor of 2 difference between SAT and Thetae_sfc trends. Thetae_sfc increased more uniformly (than SAT) between the midlatitudes of the southern hemisphere and the northern hemisphere, revealing the global nature of the heating added by greenhouse gases (GHGs). Trends in heat extremes and extreme precipitation are correlated strongly with the global/tropical trends in Thetae_sfc. The tropical amplification of Thetae_sfc is as large as the arctic amplification of SAT, accounting for the observed global positive trends in deep convection and a 20% increase in heat extremes. With unchecked GHG emissions, while SAT warming can reach 4.8°C by 2100, the global mean Thetae_sfc can increase by as much as 12°C, with corresponding increases of 12°C (median) to 24°C (5% of grid points) in land surface temperature extremes, a 14- to 30-fold increase in frequency of heat extremes, a 40% increase in the energy available for tropical deep convection, and an up to 60% increase in extreme precipitation.

## Significance

The Earth has warmed by 1.2 ± 0.1 °C since the preindustrial era. The most common metric to measure the ongoing global warming is surface air temperature since it has long and reliable observational records. However, surface air temperature alone does not fully describe the nature of global warming and its impact on climate and weather extremes. Here we show that surface equivalent potential temperature, which combines the surface air temperature and humidity, is a more comprehensive metric not only for the global warming but also for its impact on climate and weather extremes including tropical deep convection and extreme heat waves. We recommend that it should be used more widely in future climate change studies.

The planet has warmed by 1.2 ± 0.1 **°**C from its preindustrial values (1–3), three quarters of which occurred in the past 40 y mostly due to anthropogenic emissions of greenhouse gases (GHGs). Some extreme weather events in recent years have been attributed to this anthropogenic warming (4). Attributing weather extremes to global warming is one of the major new developments in climate science (4). In fact, the *Bulletin of the American Meteorological Society* concluded in 2017 (5) that “we’re experiencing new weather, because we’ve made a new climate.” Weather extremes connect global warming directly to human health. More than 80% of the world’s land regions are experiencing increased heat extremes due to human-induced GHG forcing (1). According to United Nations data (6), from 1995 to 2015, 90% of disasters were weather related, resulting in about 606,000 lives lost and 4.1 billion people injured and left homeless, among other adverse impacts. The number of weather disasters doubled during 2005 to 2014 compared with 1985 to 1994, while global warming is happening faster than the warming projected even a few years ago (7).

Surface air temperature (SAT) has been a widely accepted climate variable used to evaluate global warming. However, SAT by itself does not fully reflect the effect of global warming on climate and weather extremes. The radiative heating of the surface and the atmosphere by GHGs forcing increases both air temperature, i.e., atmospheric internal energy, and humidity, i.e., latent energy. Two factors contribute to the humidity increase: 1) increase in surface temperature, SAT, by GHGs increases evaporation of water vapor from the surface and 2) a warmer atmosphere can hold more water vapor because of the exponential increase of saturation vapor pressure, e_s,_ with temperature. Both the increase in surface evaporation with SAT and the increase in e_s_ result from the Clausius–Clapeyron equation for saturation vapor pressure, e_s_, which dictates that e_s_ increases by about 6 to 15% (depending on the temperature; *SI Appendix*, section 1) per degree of warming.

One of the most robust findings of most if not all climate model studies is that increase in SAT leads to increase in humidity (1). This was shown first in a climate model study (8). It was subsequently shown with humidity and temperature observations (9, 10) that spatial (latitudinal) as well as temporal (seasonal to interannual) variations in the observed humidity also follow the temperature dependence dictated by the Clausius–Clapeyron equation. Thus, the humidity increase with temperature, known as the water vapor–temperature feedback, is a fundamental property of the climate system, and it influences climate change in three major ways:

First, it amplifies the warming by a factor of 1.5 to 2 (8, 10, 11). The humidity increase with temperature is just as important, if not more so, in determining the magnitude of global warming. Since water vapor is the dominant GHG in the atmosphere, the water vapor–temperature feedback amplifies the water vapor greenhouse effect, which in turn amplifies the warming significantly.

Second, the latent heat released in the atmosphere, which is a primary driver of tropical convection and the general circulation, also increases with the warming because of the exponential increase of saturation vapor pressure with temperature.

Third, as shown in more detail in this study, the humidity increase with global warming and the associated increase in latent energy of the atmosphere play a major role in determining weather extremes. They have also led to more frequent and stronger extreme events such as heat waves, hurricanes, convection storms, and flash floods (12–15).

In summary, the increase in humidity and latent energy with temperature is a fundamental property of both climate and climate change. Thus, a more comprehensive metric of climate change is the change in the surface equivalent potential temperature (Thetae_sfc), which is an integrated metric of both temperature and humidity changes. When multiplied by *C_P_*, the specific heat of air at constant pressure (*SI Appendix*, section 2), Thetae_sfc is equivalent to a fundamental energy quantity called moist enthalpy. At the surface, moist enthalpy is also referred to as moist static energy. We prefer the variable Thetae_sfc (instead of enthalpy or moist static energy) since it can be readily compared with SAT in temperature units.

Thetae_sfc is also a governing parameter for atmospheric convective instability, the depth of penetration of convective clouds, the depth of the tropical troposphere, the severity of heat waves, and the onset of tropical rainfall, and it has been widely used to predict the location of monsoon rainfall (16, 17).

Both SAT and humidity increases also play a major role in how the warming translates into public health effects. For example, a useful predictor of health impacts associated with heat waves is the surface wet-bulb globe temperature (WBGT), which is a measure of heat stress a human body can endure (18–20). A simplified WBGT that accounts for both the effect of temperature and humidity (*Methods*) is highly correlated (99%) with the Thetae_sfc (*SI Appendix*, Fig. S1).

Despite the importance of Thetae_sfc, to our knowledge, it has not received much attention outside the meteorology community; only one previous study examined its historical change during 1973 to 2003 based on observational datasets (21). Here we examine global warming and its related weather and climate extremes through the lens of Thetae_sfc, a more comprehensive thermodynamic measure of global warming.

## Results

Thetae_sfc (see *Methods* for the calculation) is derived from independent datasets, including observations from the UK Met Office Hadley Centre and the Climatic Research Unit at the University of East Anglia (HadCRU) (22–24), National Center for Environmental Prediction/National Center for Atmospheric Research reanalysis 1 (NCEP1) (25), European Centre for Medium-Range Weather Forecasts (ECMWF) reanalysis v5 (ERA5) (26), Modern-Era Retrospective Analysis for Research and Applications version 2 (MERRA2) (27), and the Japanese 55-year reanalysis (JRA55) (28) (see *Methods* for additional details of the datasets). We focus on the common period of 1980 to 2019 since most reanalysis data are available during this period and the bulk of the observed SAT and Thetae_sfc trends since the preindustrial era occurred during this period (*SI Appendix*, Fig. S2). Among them, HadCRU is derived directly from observations of SAT and surface humidity. The other four datasets are reanalysis products that assimilate observed SAT as well as observed atmospheric temperature and pressure in dynamical models to simulate water vapor and other variables. NCEP1 and MERRA2 are reanalysis datasets from the United States, ERA5 is a recent European reanalysis dataset, and JRA55 is a Japanese reanalysis dataset.

**Fig. 1.**
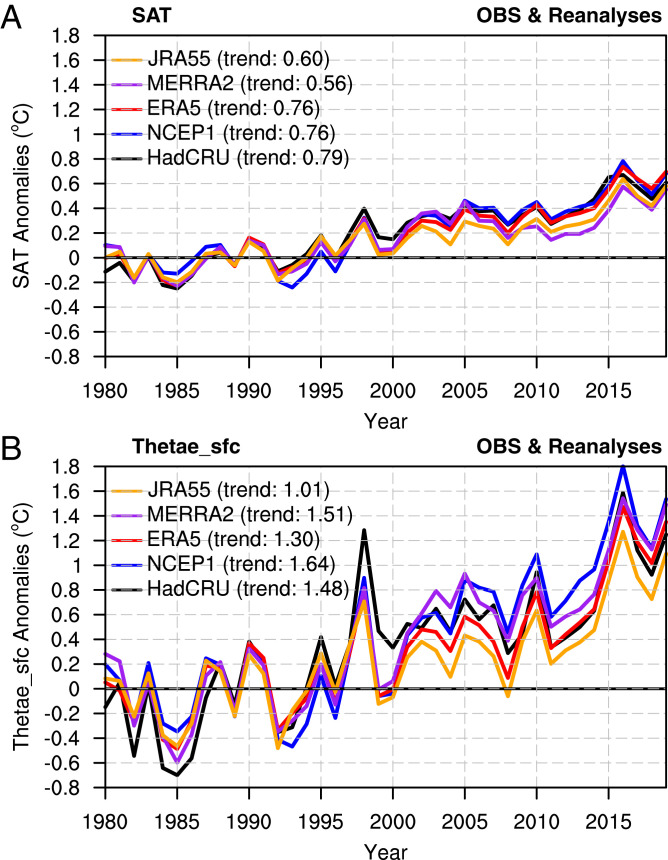
Time evolution of global mean SAT (°C) and Thetae_sfc (°C). Annual mean time series of (*A*) SAT anomalies and (*B*) Thetae_sfc anomalies from HadCRU (black line), NCEP1 (blue line), ERA5 (red line), MERRA2 (purple line), and JRA55 (orange line). The anomalies are with respect to the 1980 to 1999 mean. The numbers in HadCRU and reanalysis legends show the total warming during 1980 to 2019.

The four reanalysis datasets have similar time evolution in SAT and Thetae_sfc to the observations (HadCRU). The observations have a trend of 0.79 °C over the 40-y period, while the trends range from 0.56 to 0.76 °C in the four reanalysis datasets (Fig. 1*A*). The magnitude of the Thetae_sfc trends is significantly different from that of SAT. Thetae_sfc has much larger temporal variations than SAT, and the linear trend (1.48 °C) is roughly double that of SAT (0.79 °C) in the observations. Similar magnitudes have also been captured in the four reanalysis datasets. Thetae_sfc variations consist of contributions from temperature and moisture (${\text{θ}}_{\text{e}}\_T$ and ${\text{θ}}_{\text{e}}\_M$, respectively; Eqs. **2** and **3** in *Methods*), which contribute about equally to the Thetae_sfc trends (*SI Appendix*, Fig. S3).

**Fig. 2.**
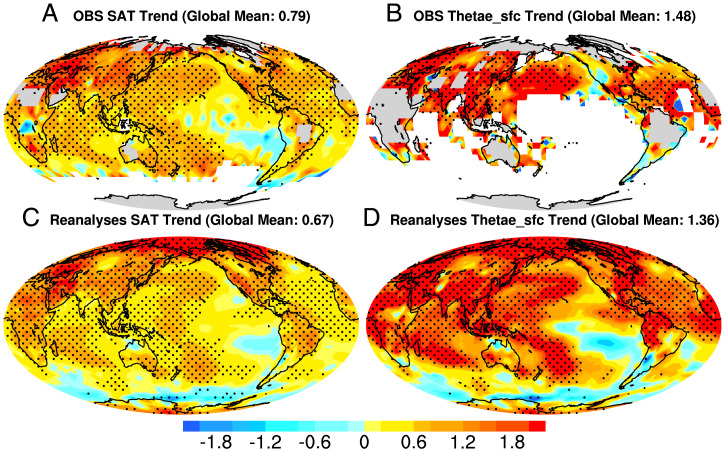
Global map of warming pattern in SAT and Thetae_sfc during 1980 to 2019. Linear trend of (*A*) SAT in observations (OBS), (*B*) Thetae_sfc in OBS, (*C*) SAT in reanalyses, and (*D*) Thetae_sfc in reanalyses. The trend has been normalized by the global mean value shown in the title of each panel. Results in the reanalyses are obtained by averaging over all four reanalysis datasets (NCEP1, ERA5, MERRA2, and JRA55).

The geographical pattern of warming in terms of the SAT trend during 1980 to 2019 shows three prominent features: larger warming in northern hemisphere (NH) than southern hemisphere (SH), much larger midlatitude to polar warming than tropical warming in NH, and enhanced warming over land relative to oceans (Fig. 2 and *SI Appendix*, Fig. S4), all of which have been well documented (1, 2). However, all of these three warming contrasts are significantly weakened when measured in Thetae_sfc (Fig. 2). In other words, the Earth is heated more uniformly when measured by Thetae_sfc. In Table 1, we quantify these differences of SAT and Thetae_sfc by showing the warming ratios between NH and SH, between land and ocean, and between tropical and NH polar region. The warming trend in NH and land is roughly twice (2.19 and 1.91, respectively) as much as that in SH and in oceans in the observations measured by SAT. However, the NH–SH contrast and land–ocean contrast in Thetae_sfc trend are reduced to 1.58 and 1.24, respectively. When measured by SAT, the tropical warming is only 31% of the NH polar warming, known as the Arctic amplification phenomenon, but Thetae_sfc trend in the tropics is more than half of that in the Arctic. All these features are well captured in the reanalysis datasets and climate models forced by observed sea surface temperature. Note that the NH/SH ratio in observations is quite different from reanalysis datasets and AMIP (Atmospheric Model Intercomparison Project) simulations. This is likely due to more missing values in SH than NH in observations. For future changes, the tropical amplification of the Thetae_sfc trends is nearly comparable to the polar amplification of SAT in NH (*SI Appendix*, Table S1).

**Table 1.** The ratio of linear trend of SAT and Thetae_sfc in the observation, average of four reanalysis datasets, and average of two AMIP runs during 1980 to 1999

Data	Land/ocean ratio		NH/SH ratio		Tropics/NH polar ratio	
	SAT	Thetae_sfc	SAT	Thetae_sfc	SAT	Thetae_sfc
OBS	1.91	1.24	2.19	1.58	0.31	0.53
REA	2.17	1.68	2.94	2.80	0.24	0.42
AMIP	2.08	1.59	2.39	2.11	0.34	0.79

Partitioning the Thetae_sfc trends in terms of the temperature trends and moisture trends shows that the tropical and subtropical Thetae_sfc trend is mainly contributed by the moisture component (${\text{θ}}_{\text{e}}\_M$; *SI Appendix*, Fig. S5), which is due to the much larger increase of moisture. Most of the tropical and subtropical Thetae_sfc increase occurs over ocean and tropical forests, including Amazon, Congo, and Maritime Continent, where there is abundant moisture.

**Fig. 3.**
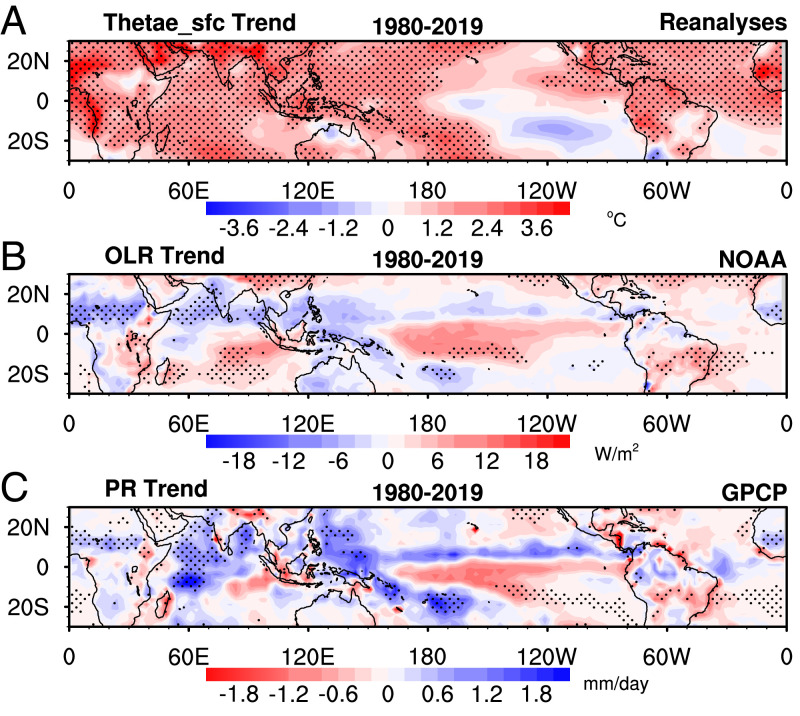
Changes of Thetae_sfc in the tropics and its implication for convection. The spatial pattern of linear trend of annual mean (*A*) Thetae_sfc (°C) from the mean of four reanalysis datasets (NCEP1, ERA5, MERRA2, and JRA55), (*B*) OLR (W m^−2^) from NOAA, and (*C*) precipitation (mm d^−1^) from GPCP during 1980 to 2019. The stippled areas indicate that the linear trend is significant at the 5% level.

Because surface humidity observations are not regionally uniform, particularly over the oceans and in the SH, we examine correlative satellite data (Fig. 3) to confirm the spatial patterns of the Thetae_sfc trends. In addition, comparison with cloudiness and precipitation data from satellites reveals their direct link with the trend patterns of Thetae_sfc. More specifically, the Thetae_sfc trends illustrated by both the observations and reanalysis datasets are consistent with the trends in convection and cloud depth revealed by independent satellite datasets (Fig. 3). Corresponding to the maximum increase of nearly 4 °C in Thetae_sfc over tropical marine locations and tropical forests, there is a decrease in outgoing longwave radiation (OLR) at the top of the atmosphere due to increase in cloud top altitudes from deeper convection (Fig. 3*B*), an increase in precipitation (Fig. 3*C*), and an increase in cloud radiative forcing (*SI Appendix*, Fig. S6 *A* and *B*). These change patterns are well captured by climate models forced with observed sea surface temperatures (*SI Appendix*, Figs. S6 *C–F* and S7). The trends in convective available potential energy (CAPE) and the altitudes of convective cloud tops estimated from the reanalysis data (*SI Appendix*, Fig. S8) are consistent with the changes in OLR, cloud radiative forcing, and precipitation.

Besides the annual mean precipitation change, during the satellite period, another significant change in tropical rainfall is the emergence of its seasonal delay over land, with decreased rainfall in the spring and increased rainfall in the fall (29) (*SI Appendix*, Fig. S9*A*). The delayed onset of tropical rainfall annual cycle is a robust feature under global warming and is found to be linearly related to global warming (30–32). However, it is not well represented by the annual cycle of SAT trend (*SI Appendix*, Fig. S9*B*). On the other hand, Thetae_sfc trend shows a clear correspondence to the rainfall delay, with cooling during spring and warming during fall (*SI Appendix*, Fig. S9*C*). In short, the Thetae_sfc trends are not only consistent among different observations and observationally constrained products and model simulations, but they also capture the global hydrological cycle changes much better than the SAT trends.

**Fig. 4.**
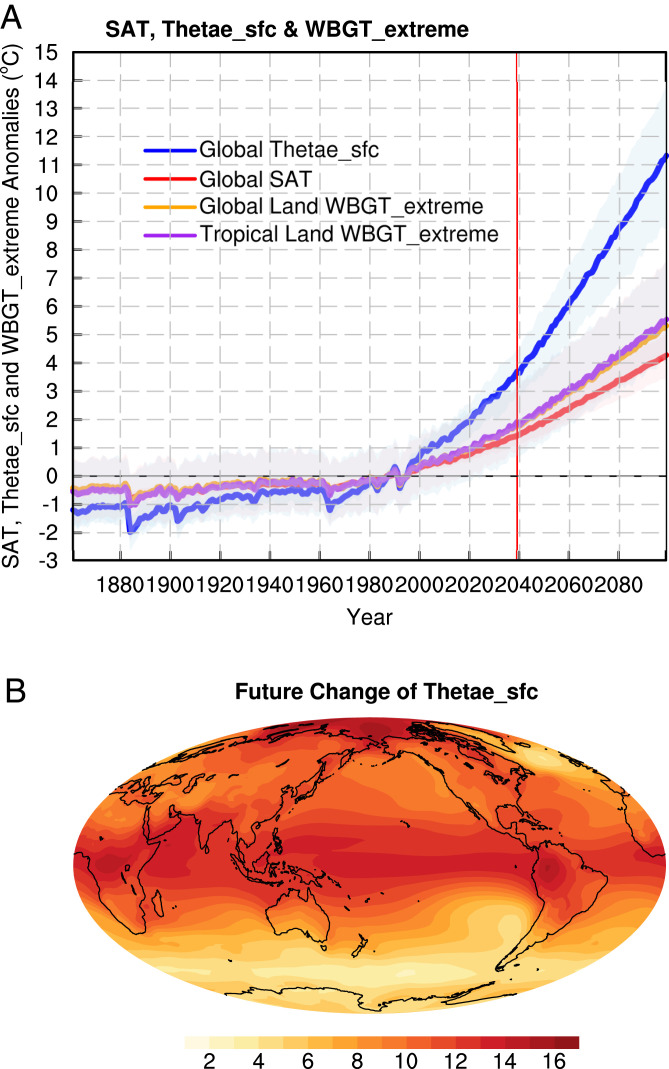
Future changes of SAT and Thetae_sfc. (*A*) Annual mean time series of SAT (red line; °C), Thetae_sfc (blue line; °C), extreme WBGT over global land (orange line; °C), and extreme WBGT over tropical land (purple line; °C) anomalies relative to the 1980 to 1999 mean and (*B*) the future change patterns of ${\text{θ}}_{\text{e}}$ between future climate (2080–2099) and current climate (1980–1999). The shading indicates intermodel spread among 20 CMIP5 models.

With unchecked emissions of GHGs, the future changes of Thetae_sfc can be even more pronounced. Here we examine the Thetae_sfc changes under the Representative Concentration Pathways 8.5 (RCP85) scenario based on 20 Coupled Model Intercomparison Project phase 5 (CMIP5) models (*SI Appendix*, Table S2). The ensemble average of SAT increases almost linearly with time, to 2 °C higher than the SAT for the preindustrial era by 2039, and 4.8 °C higher at the end of the 21st century (Fig. 4*A*). The extreme WBGT, which is defined as the 95th percentile of daily WBGT temperature (*Methods*), in both tropical land and global land increases by close to 6 °C by the end of the century relative to the preindustrial era. Thetae_sfc increases at an increasingly faster rate, reaching 4.9 and 12.5 °C higher than the preindustrial era Thetae_sfc by 2039 and the end of the 21st century, respectively. Similar to the linear trend pattern during 1980 to 2019 (Fig. 2*A*), future SAT warming also exhibits polar amplification and enhanced land–sea contrast (*SI Appendix*, Fig. S10*A*). In contrast, Thetae_sfc warming is more uniform between land and ocean and subject to both polar and tropical amplifications (Fig. 4*B*), with the former dominated by the temperature component ${\text{θ}}_{\text{e}}\_\text{T}$ and the latter dominated by the moisture component ${\text{θ}}_{\text{e}}\_\text{M}$ (*SI Appendix*, Fig. S10). The regional changes by the end of the century can be as much as 16 °C in both the tropics and NH polar regions (Fig. 4*B*).

## Implications for Extreme Weather

Examining climate change with the Thetae_sfc lens provides more insights into the links between warming and extreme weather. With unchecked emissions, global mean Thetae_sfc can increase by 12.5 **°**C in 2100 relative to the preindustrial era. What do the huge increases in Thetae_sfc mean for extreme weather events? We describe three types of extremes below.

### Deep Convection and Tropical Dynamics.

**Fig. 5.**
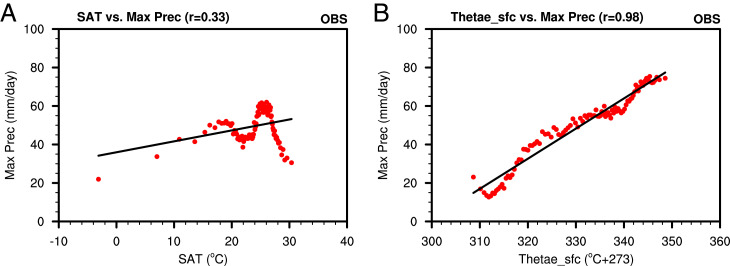
Relationship between annual maximum precipitation and annual mean (*A*) SAT and (*B*) Thetae_sfc over tropical land (30°S to 30°N) during 1980 to 2019. The precipitation data are from NOAA Climate Prediction Center gridded, gauge-based daily observations, and SAT and Thetae_sfc are from ERA5 reanalysis. Each point represents a bin average of data from all land grid points in the latitude belt. The correlation coefficient is given in the parentheses at the top of each frame.

Thetae_sfc has already been shown to be a good metric for deep convection (Fig. 3), as also demonstrated by much higher correlation with precipitation and CAPE than SAT (*SI Appendix*, Figs. S11 and S12), especially over land regions. More importantly, Thetae_sfc is much more tightly linked to extreme precipitation than SAT from both observations and climate model simulations over land (Fig. 5 and *SI Appendix*, Fig. S13), where most of the human activities take place. By the end of the century, with unchecked global warming, the intensity of model projected extreme precipitation can increase by 40 to 60% relative to current extreme precipitation (*SI Appendix*, Fig. S14). As extreme precipitation has a profound societal impact, this once again demonstrates the importance of using Thetae_sfc as a better metric for evaluating the impacts of global warming. Deep convection and the associated mesoscale and cirrus anvil cloud systems are the primary mechanisms by which latent heat is released to drive the large-scale tropical atmospheric circulation. They are linked to monsoon circulation, tropical hurricanes, and extensive high-level cirrus clouds (33, 34). With increased Thetae_sfc, the atmosphere is more convectively unstable, as measured by CAPE, and the maximum altitude a convective parcel from the surface can reach, a proxy for cloud top altitudes of convective clouds (35, 36), is higher (*SI Appendix*, Fig. S8). The tropical average (30° S to 30° N) increases of CAPE and convection top height are 47 J/kg and 102 m during 1980 to 2019, respectively. These represent a 9% increase for CAPE and 1.4% increase for convection top height, relative to the climatological values of 1980 to 1999. By 2100, the 16 °C increase in tropical Thetae_sfc can increase CAPE by more than 200 J/kg, over 30% increase relative to 1980 to 1999 (*SI Appendix*, Fig. S15). The increase of CAPE in both observations and projected future climate can partly explain the increased rainfall extremes and severe thunderstorms (37–40).

### Extreme Heat Waves.

**Fig. 6.**
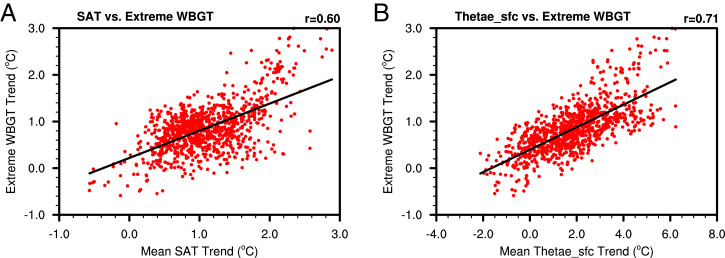
The extreme WBGT trend is highly correlated with mean Thetae_sfc trend over tropical land (30° S to 30° N) during 1980 to 2019 in ERA5. The scatter plots are between (*A*) mean SAT trend and extreme WBGT trend and (*B*) mean Thetae_sfc trend and extreme WBGT trend. Each data point represents one location.

Heat waves, as measured by extremes (hottest 5% in daily mean values) in WBGT, are highly correlated with the tropical mean Thetae_sfc (*SI Appendix*, Fig. S17). In the past 20 y, the latest decade is warmer than the prior decade. Correspondingly, there is more extreme WBGT and higher tropical mean Thetae_sfc than the prior decade. The relationships between WBGT and tropical mean Thetae_sfc are very well captured by GCMs for both current and projected future climates (*SI Appendix*, Fig. S16 *B* and *C*). For every 1 °C change in tropical mean Thetae_sfc, there is a 0.4 to 0.5 °C change in extreme WBGT, as inferred from the slopes in *SI Appendix*, Fig. S16. The ratio of the trend in WBGT extremes over land to that of the global mean Thetae_sfc is also highly concentrated at 0.5 for both current and future climates, more so for the latter (*SI Appendix*, Fig. S17). During 1980 to 2019, the relationship with the extreme WBGT trend is considerably higher in the mean Thetae_sfc trend than the mean SAT trend (with a correlation of 0.73 vs. 0.59; Fig. 6). As the global mean Thetae_sfc increases (Fig. 3*A*), future heat extremes become more severe (*SI Appendix*, Figs. S18 and S19). The extreme summer daily mean WBGT in different parts of the world (India, northern China, North America, and Europe) can increase by as much as 6 °C by the end of the 21st century compared with the heat extremes in the current climate. Presently, extreme WBGT in many parts of the world (India, eastern China, eastern United States, and northern Australia; *SI Appendix*, Fig. S18) has already reached 32 °C, an extreme level of risk for outdoor activities (20). An increase in WBGT extremes by another 6 °C beyond the current extremes would be debilitating, particularly for the vulnerable population of 3 billion or more and for many ecosystems, as extreme WBGTs exceeding 35 °C (approximately equal to human skin temperature) pose hazardous levels of risks to human health (20). The occurrence frequency of WBGT extremes exceeding 35 °C increases by 14-fold over India and southern China, 30-fold over northern China, 22-fold over North America, and 23-fold over Europe by 2100 (*SI Appendix*, Fig. S19).

## Concluding Remarks

Thetae_sfc is often known as a measure of convective instability and the potential for atmospheric convection, but it has not yet been used to gauge global warming. In this study we showed that Thetae_sfc can be used as a metric, complementary to the widely used SAT, to measure global warming and its impact. SAT has the largest signal of global warming in high latitudes due to polar amplification. On the other hand, there are pronounced increases of Thetae_sfc in both the tropics and high latitudes as the Earth becomes warmer in observations and climate projection, owing to its inclusion of both temperature and moisture changes. The trends in Thetae_sfc match the trends in convection and cloud radiative forcing. More importantly, trends in mean Thetae_sfc are highly correlated with heat wave extremes both in the past and in future climate projection by models. Thetae_sfc is also tightly linked to extreme precipitation in both observations and global climate models. These findings suggest that Thetae_sfc complements SAT as a comprehensive metric of weather extremes caused by global warming and should be used more widely in future climate change studies.

## Methods

### Observational and Reanalysis Datasets.

In this study, monthly SAT from HadCRUT4 (HadCRU Temperature data version 4) (22) is used to represent the observed SAT, while monthly surface specific humidity from Hadley Centre gridded global surface humidity dataset (HadISDH).blend version 1.1.1.2020f (23, 24) together with SAT are used to calculate the Thetae_sfc (${\text{θ}}_{\text{e}}$) based on the method shown in *Calculation of Surface Equivalent Potential Temperature*. We refer to the SAT and ${\text{θ}}_{\text{e}}$ as HadCRU, as both SAT and specific humidity are developed by the Climatic Research Unit (University of East Anglia) and the Hadley Centre (UK Met Office). We also use monthly SAT, surface specific humidity, and surface pressure from NCEP1 (25), ERA5 (26), MERRA2 (27), and JRA55 (28) to calculate ${\text{θ}}_{\text{e}}$. For ${\text{θ}}_{\text{e}}$ calculation in HadCRU, surface pressure is from NCEP1. Daily SAT and surface specific humidity from ERA5 during 1980 to 2019 are also used. The OLR from National Oceanic and Atmospheric Administration (NOAA) is used for the period of 1980 to 2019, which combines several satellite datasets (41). The precipitation from Global Precipitation Climatology Project (GPCP) is used for the period of 1980 to 2019 (42). The shortwave and longwave cloud radiative forcing from International Satellite Cloud Climatology Project (ISCCP) are used for the period 1984 to 2007 (43). The reference period is chosen as 1980 to 1999 throughout the study to calculate the monthly anomalies, unless stated otherwise.

### Model Simulations.

To obtain the future changes of ${\text{θ}}_{\text{e}}$, monthly mean outputs of SAT, surface specific humidity, and surface pressure from historical and RCP8.5 simulations of 20 CMIP5 (44) models are used (*SI Appendix*, Table S2). Except the Community Earth System Model version 1 (CESM1) simulation CESM1-CAM5, daily mean outputs of SAT, surface specific humidity, and surface pressure from historical and RCP8.5 simulations of these 20 models are also used. To assess the models’ ability in capturing the current linear trends of convection, AMIP-type simulations from two models (CCSM4 and HadGEM2-A) are also used.

### Calculation of Surface Equivalent Potential Temperature.

We use the following simple formula based on Taylor expansion to calculate ${\text{θ}}_{\text{e}}$ (45):

[1] $$\theta_{e}\approx \left(T+\frac{L_{v}}{C_{p}}r\right){\left(\frac{p_{0}}{p_{s}}\right)}^{\frac{R_{d}}{C_{p}}}.$$

Here T is the SAT Tas; $L_{v}$ is latent heat of vaporization (here it is taken as 2,500 kJ kg^−1^); $C_{p}$ is specific heat of dry air at constant pressure (here it is taken as 1,005.7 J K^−1^ kg^−1^); r is the mixing ratio, which is roughly equal to specific humidity q (it should be $r=\frac{q}{1-q}$ to be exact); $p_{0}$ is reference pressure (1,000 hPa); $p_{s}$ is surface pressure; and $R_{d}$ is specific gas constant for air (287.04 J K^−1^ kg^−1^). We can also divide $\theta_{e}$ into two components: one is linearly related to temperature ($\theta_{e}\_T$), and the other is related to moisture ($\theta_{e}\_M$). The two components are expressed as

[2] $$\theta_{e}\_T\approx T{\left(\frac{p_{0}}{p_{s}}\right)}^{\frac{R_{d}}{C_{p}}},$$

[3] $$\theta_{e}\_M\approx \frac{L_{v}}{C_{p}}r{\left(\frac{p_{0}}{p_{s}}\right)}^{\frac{R_{d}}{C_{p}}}.$$

Although $\theta_{e}\_M$ is quite small compared to $\theta_{e}\_T$ in terms of absolute values (50 K vs. 300 K), here what matters is the temporal changes, which are comparable. According to the Clausius–Clapeyron equation and the relationship between saturation specific humidity and saturation vapor pressure, we can easily obtain the relationship between r and T as follows:

[4] $$r=\frac{\epsilon Re_{s0}}{p}e^{\frac{L_{v}}{R_{\text{v}}}(\frac{1}{T_{0}}-\frac{1}{T})}.$$

Here ϵ is the ratio of the gas constants for dry air and water vapor (287 and 461 J K^−1^ kg^−1^, respectively), R is relative humidity, $e_{s0}$ is vapor pressure at reference temperature $T_{0}$ (273.15 K), and $R_{v}$ is gas constant for water vapor (461 J K^−1^ kg^−1^). Hence, $\theta_{e}\_M$ can also be written as

[5] $$\theta_{e}\_M\approx \frac{L_{v}\epsilon Re_{s0}}{C_{p}p}e^{\frac{L_{v}}{R_{v}}(\frac{1}{T_{0}}-\frac{1}{T})}{\left(\frac{p_{0}}{p_{s}}\right)}^{\frac{R_{d}}{C_{p}}}.$$

In this form, $\theta_{e}\_M$ is nonlinearly related to SAT and increases faster with temperature at warmer temperatures.

### WBGT.

The WBGT is a measure of heat stress in direct sunlight; it has been used by several western countries to provide guidance for military training and heavy labor activities in summer. The definition of WBGT involves sunlight incidence, wind speed, temperature, and humidity, some of which are not available in archived observational data. Therefore, we use the simplified form from ref. 20:

[6] WBGT=0.567T+0.393e+3.94,

where T is temperature in Celsius and *e* is the vapor pressure of the air in hPa. The WBGT values at 28, 32, and 35 °C correspond to high, very high, and extreme risk to health (20).

## Supplementary Material

Supplementary File

## Data Availability

All data used in this study are publicly available. The HadCRUT4 dataset can be downloaded from the following website: https://crudata.uea.ac.uk/cru/data/temperature/#datdow. The HadISDH.blend version 1.1.1.2020f dataset can be downloaded from the following website: https://www.metoffice.gov.uk/hadobs/hadisdh/downloadblend1112020.html. The NCEP1 reanalysis dataset is available at https://psl.noaa.gov/data/gridded/data.ncep.reanalysis.html. The ERA5 reanalysis dataset can be downloaded from the following website: https://cds.climate.copernicus.eu/#!/search?text=ERA5&type=dataset. The MERRA2 reanalysis dataset is available from https://gmao.gsfc.nasa.gov/reanalysis/MERRA-2/data_access/. The JRA55 dataset is available from https://climatedataguide.ucar.edu/climate-data/jra-55. The GPCP precipitation dataset can be downloaded from the following website: https://psl.noaa.gov/data/gridded/data.gpcp.html. The NOAA OLR dataset can be downloaded from the following website: https://www.esrl.noaa.gov/psd/data/gridded/data.interp_OLR.html. The ISCCP dataset can be downloaded from the following website: https://eosweb.larc.nasa.gov/project/isccp/isccp_table. The global climate model outputs can be obtained from the CMIP5 archive accessed through the following website: http://www.ipcc-data.org/sim/gcm_monthly/AR5/Reference-Archive.html.
