# Supplementary material for: Trends in surface equivalent potential temperature: A more comprehensive metric for global warming and weather extremes
Source: Proc Natl Acad Sci U S A. 2022 Jan 31;119(6):e2117832119. doi: 10.1073/pnas.2117832119 (PMC8833193; doi:10.1073/pnas.2117832119)
Supplement: Supplementary File [file pnas.2117832119.sapp.pdf]

Supplementary Information

Trends in Surface Equivalent Potential Temperature:

A more comprehensive metric for global warming and weather extremes

Fengfei Song[1,2], Guang J. Zhang[3*], V. Ramanathan[3*], L. Ruby Leung[2]

[1] Frontiers Science Center for Deep Ocean Multispheres and Earth System, Ocean University of China, Qingdao, Shandong, China

[2] Atmospheric Sciences and Global Change Division, Pacific Northwest National Laboratory, Richland, Washington, USA

[3] Scripps Institution of Oceanography, University of California San Diego, La Jolla, California, USA

September 28, 2021

Revised December 10, 2021

Corresponding authors:

V. Ramanathan (vramanathan@ucsd.edu) and Guang Zhang (gzhang@ucsd.edu)

Supplement Text

1. The Clausius-Clapeyron Equation

The Clausius-Clapeyron equation describes the variation of saturation vapor pressure with temperature when the liquid and vapor states of water are in equilibrium:

$$\frac{de_s}{dT} = \frac{L_v}{R_v}\frac{e_s}{T^2}$$

where e_s is saturation vapor pressure, T temperature, L_v the latent heat of vaporization and R_v gas constant of water vapor. Approximately, we can take L_v equal to 2.501x106 J kg^{-1}, and R_v equal to 461.50 J kg^{-1} K^{-1}. Thus, the relative change of saturation vapor pressure per degree temperature change ($de_s/e_s dT$) is approximately 6% at T=300 K at the surface and 15% at T=190 K near the tropopause.

2. Equivalence between the Thermodynamic Energy at the surface and Thetae

The thermodynamic energy is the sum of the thermal energy, the potential energy and the latent energy. At the surface, the potential energy is zero and hence the thermodynamic energy is determined solely by the sum of the thermal and latent energy, denoted by E. Since the bulk of solar energy is deposited at the surface, within the troposphere the total energy at the surface is larger than elsewhere in the atmosphere. Hence, globally, changes in the surface thermodynamic energy are a good measure for changes in the surface-atmosphere climate system.

The thermal energy is defined as $C_P T$, where C_P is the specific heat of the atmosphere at the surface at constant pressure; and the latent energy is defined as, Lq, where L is the latent heat of vaporization (or condensation) and q is the specific humidity. So, E= $C_P T$+ Lq, which is the same as specific moist enthalpy, and when we normalize this equation with C_P, we get: E/C_P =

$T+(L/C_P)q$. As a result, the normalized energy equation at the surface has the units of temperature. At the surface, it can easily be shown that Thetae = $\{T+(L/C_P)q\}$. The actual equation we use for Thetae is more complicated as shown in the Methods section. To give the magnitude of the two terms, over the Tropical ocean, T= 300 K {unit is degrees Kelvin= 273+ T(^{o}C)}and $(L/C_P)q$ = 50 K. Note that change in T in K is exactly equal to change in °C. So, from now onwards, change in temperature and Thetae is given in the familiar unit °C. From the above example, the latent energy of the tropical surface air is about 17% of the thermal energy. This is not the whole story however. If the relative humidity remains the same, the atmospheric surface humidity increases by about 6% per degree (K or °C) of warming. This increase is governed by the so-called Clausius-Clapeyron equation which dictates that the saturation vapor pressure of water vapor increases by about 6% (when the temperature is around 300K). In short, with fixed relative humidity, the latent energy will increase for each degree of warming by about 6%, which is (50x.06) about 3.0 °C. It follows then Thetae will increase by 4 °C with each degree warming. We will indeed show that with the observed warming of 1 °C during the last 70 years, tropical Thetae increased by about 4 °C.

Table S1 Future changes of surface air temperature (SAT) and surface equivalent potential temperature (Thetae_sfc) warming ratios between 2080-2099 and 1980-1999.

Data	Land/Ocean ratio		NH/SH ratio		Tropics/NH Polar ratio	
	SAT	Thetae_sfc	SAT	Thetae_sfc	SAT	Thetae_sfc
CMIP5	1.56	1.10	1.56	1.39	0.44	1.01

Table S2 20 CMIP5 models and experiments used in this study.

Model	HIST	RCP85	AMIP
ACCESS1-0	×	×	
ACCESS1-3	×	×	
bcc-csm1-1	×	×	
BNU-ESM	×	×	
CanESM2	×	×	
CCSM4	×	×	×
CESM1-CAM5	× (no daily data)	× (no daily data)	
CNRM-CM5	×	×	
FGOALS-g2	×	×	
GFDL-CM3	×	×	
GFDL-ESM2G	×	×	
GFDL-ESM2M	×	×	
HadGEM2-ES	×	×	× (HadGEM2-A)
inmcm4	×	×	
IPSL-CM5A-LR	×	×	
IPSL-CM5A-MR	×	×	
IPSL-CM5B-LR	×	×	
MRI-CGCM3	×	×	
NorESM1-M	×	×	
NorESM1-ME	×	×	

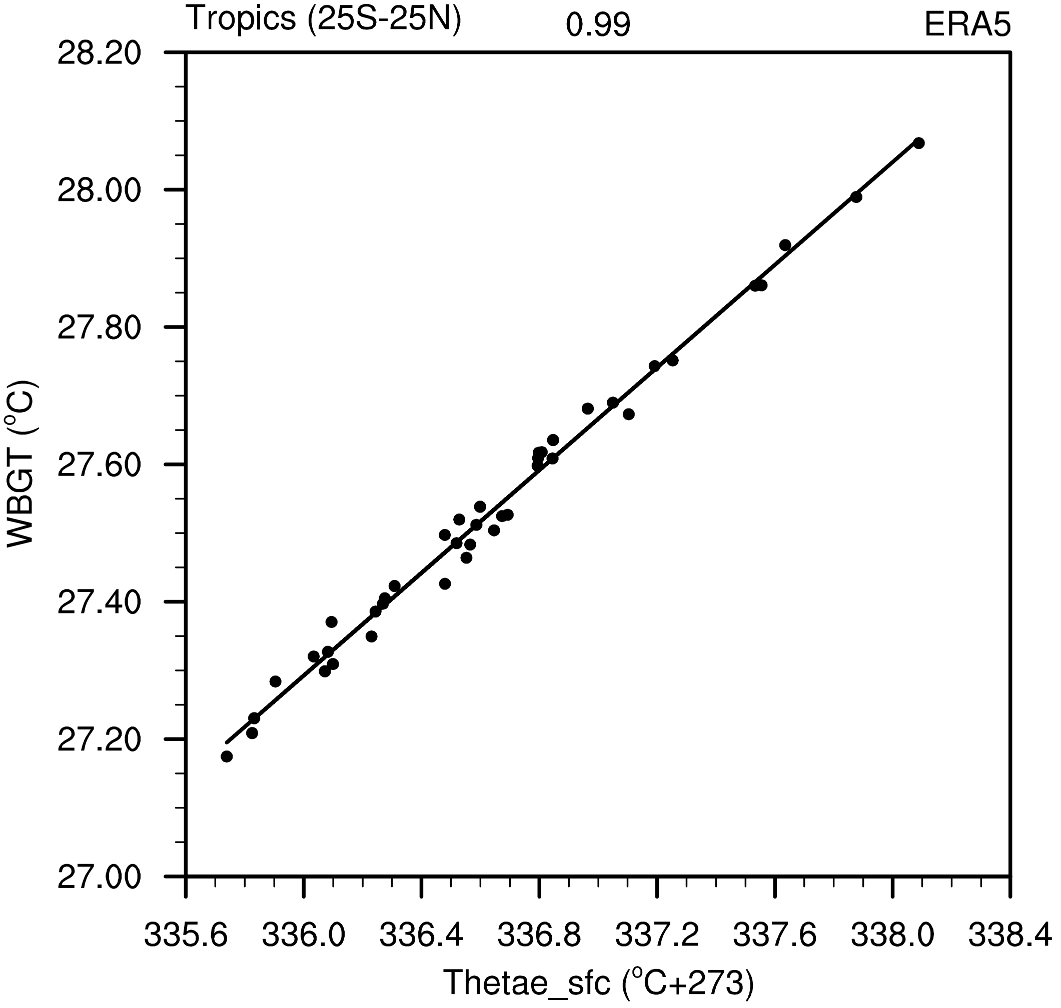

Supplementary Fig. 1 Relationship between Thetae_sfc and Wet Bulb Globe Temperature (WBGT) during 1980-2019. Each point represents an annual and tropical mean (25°S-25°N) value from the ERA5 reanalysis data. The correlation coefficient is 0.99.

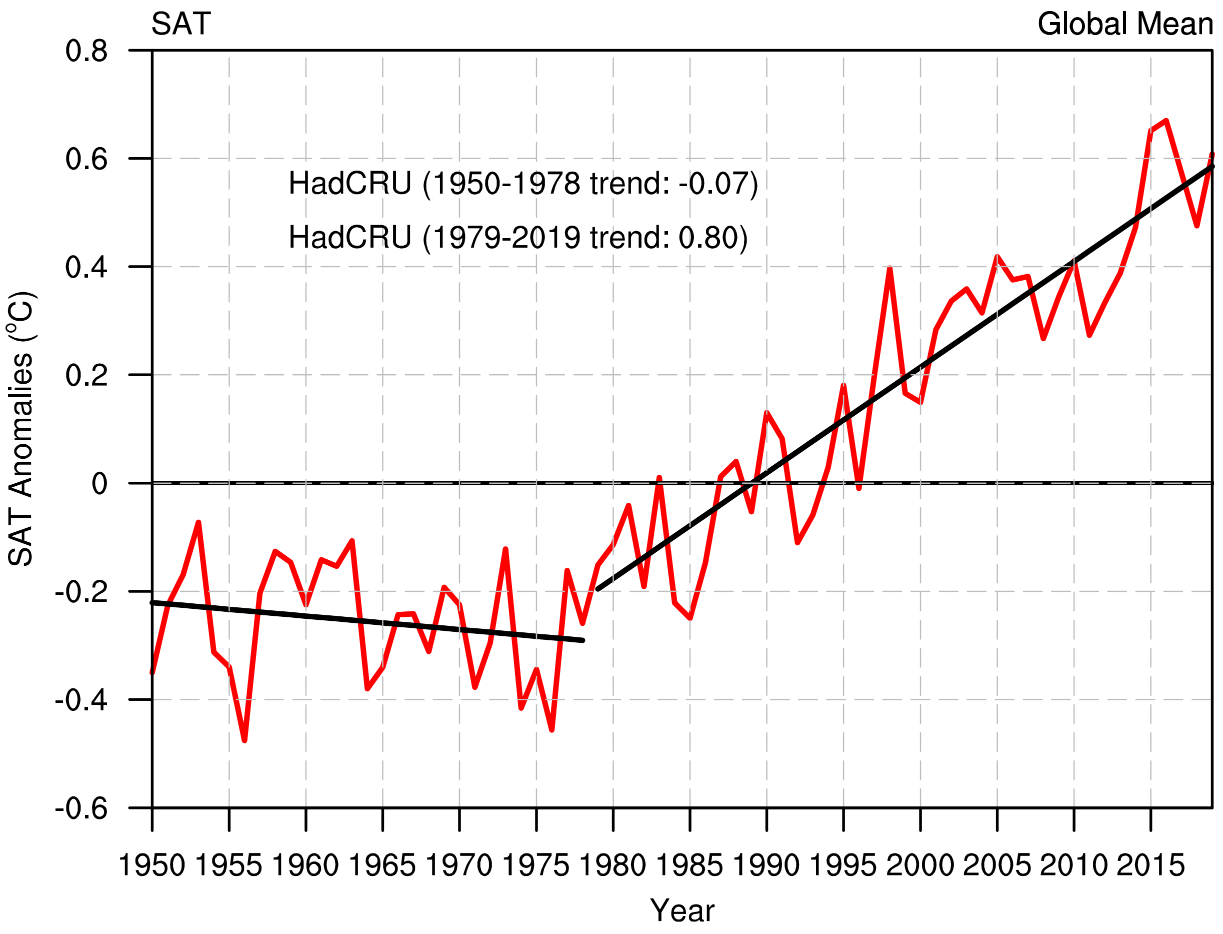

Supplementary Fig. 2 The annual-mean time series of SAT anomalies in HadCRU (°C) during 1950-2019. The reference period is 1980-1999. The linear trend of SAT during 1950-1978 is close to zero.

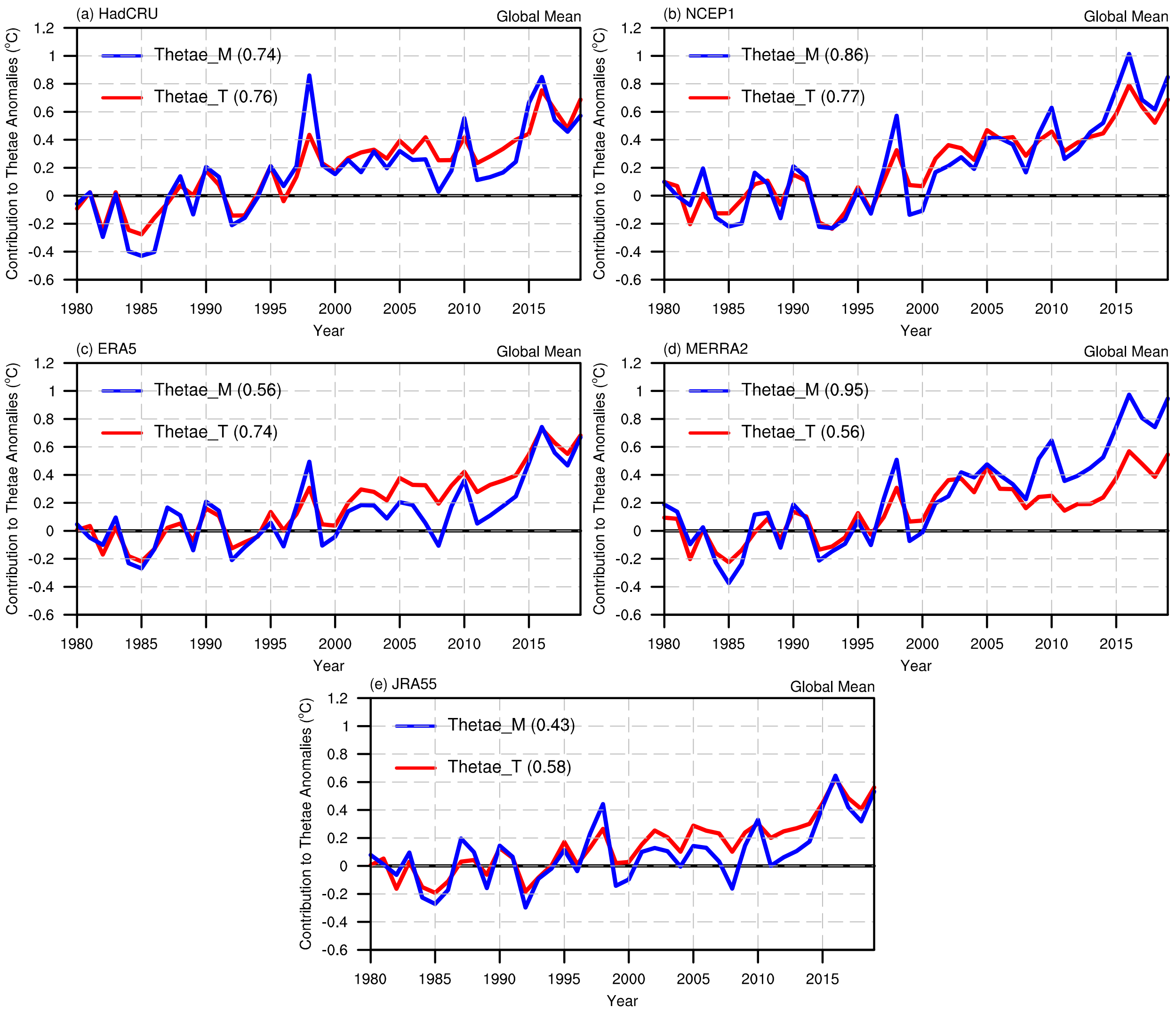

Supplementary Fig. 3 Annual-mean time series of temperature component (red line) and moisture component (blue line) of surface equivalent potential temperature (θ_e) from (a) HadCRU and (b) NCEP1, (c) ERA5, (d) MERRA2 and (e) JRA55. The reference period is 1980-1999. The numbers in the legend show the total warming during 1980-2019 (unit: ^{o}C).

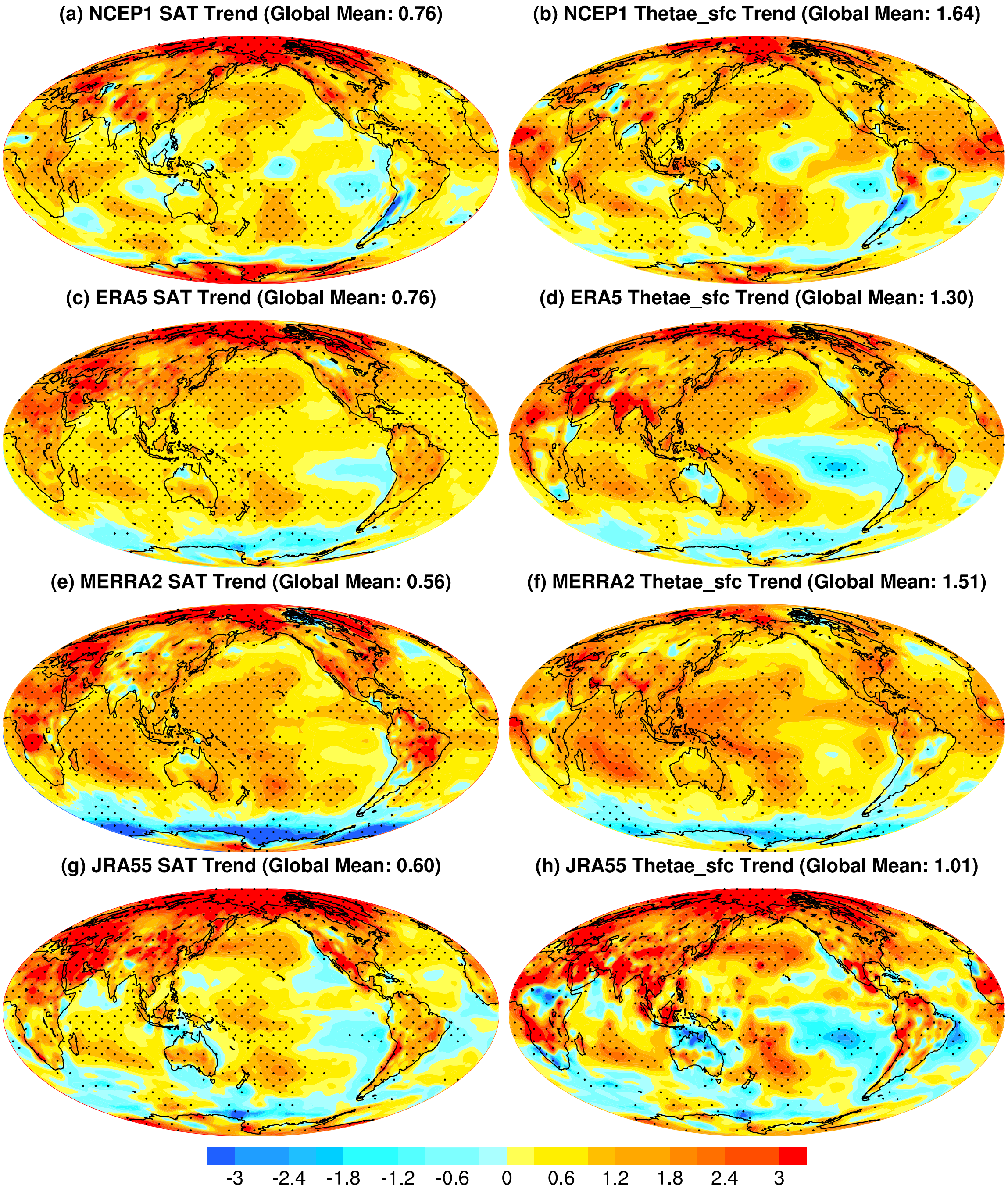

Supplementary Fig. 4 Annual-mean time series of temperature component (red line) and moisture component (blue line) of surface equivalent potential temperature (θ_e) from (a) HadCRU and (b) NCEP1, (c) ERA5, (d) MERRA2 and (e) JRA55. The reference period is 1980-1999. The numbers in the legend show the total warming during 1980-2019 (unit: ^{o}C).

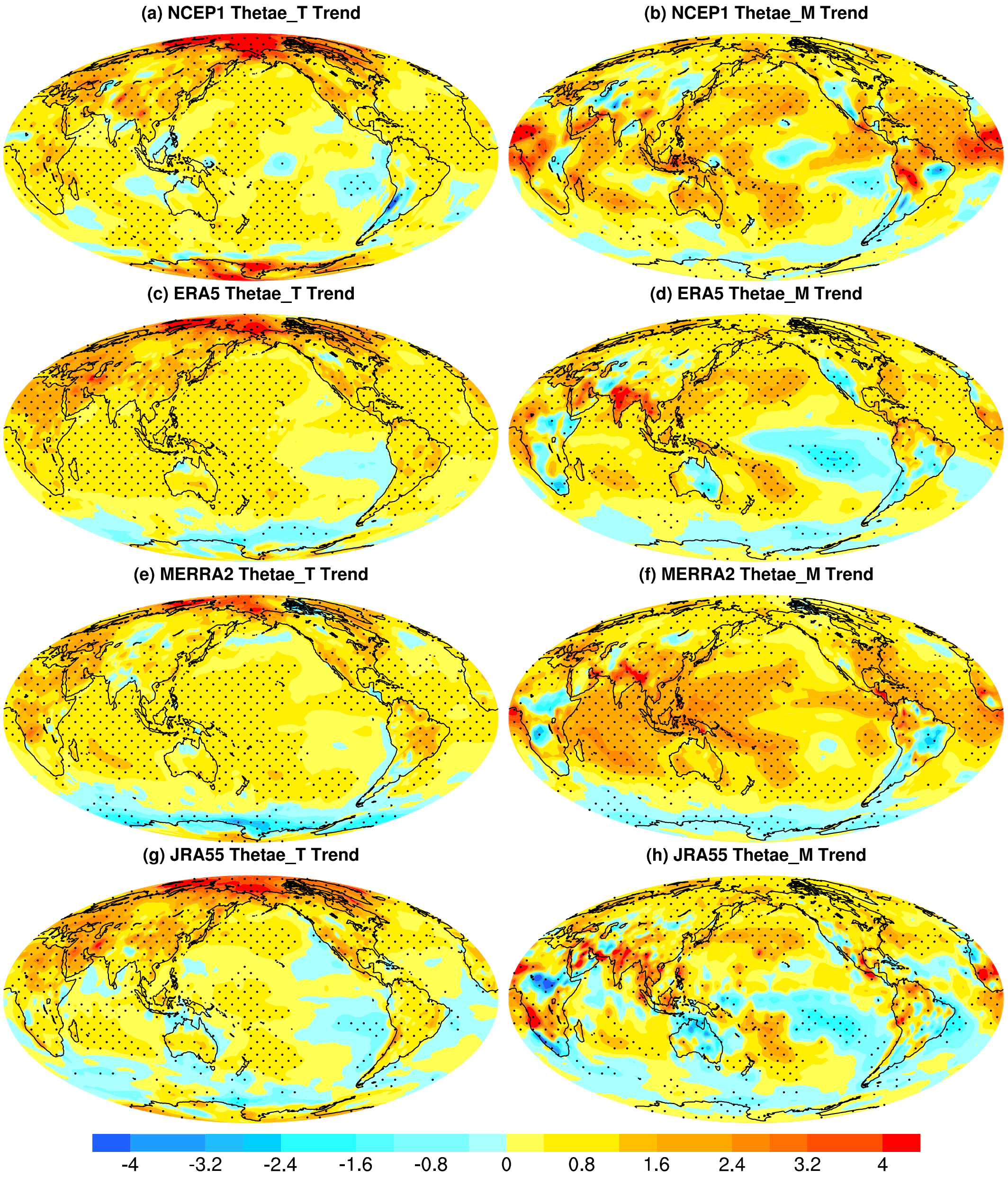

Supplementary Fig. 5 Spatial pattern of linear trend of annual-mean (left panel) temperature component (θ_e_T; unit: ^oC) and (right panel) moisture component (θ_e_M; unit: ^oC) of surface equivalent potential temperature from (a-b) NCEP1, (c-d) ERA5, (e-f) MERRA2, (g-h) JRA55 during 1980-2019. The stippled areas indicate that the linear trend is significant at the 5% level.

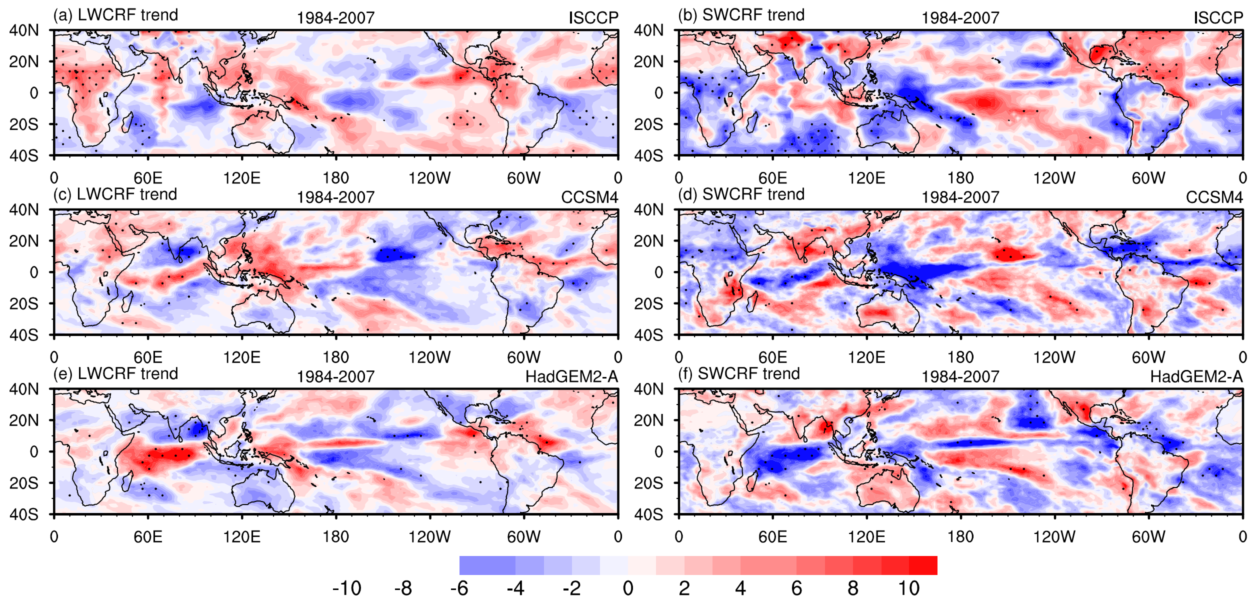

Supplementary Fig. 6 Spatial pattern of changes in annual-mean (left panel) longwave cloud radiative forcing (LWCRF; unit: W/m^2) and (right panel) shortwave cloud radiative forcing (SWCRF; unit: W/m^2) from (top panel) ISCCP, (middle panel) AMIP experiment from CCSM4 and (bottom panel) AMIP experiment from HadGEM2-A during 1984-2007. The stippled areas indicate that the change is significant at the 5% level.

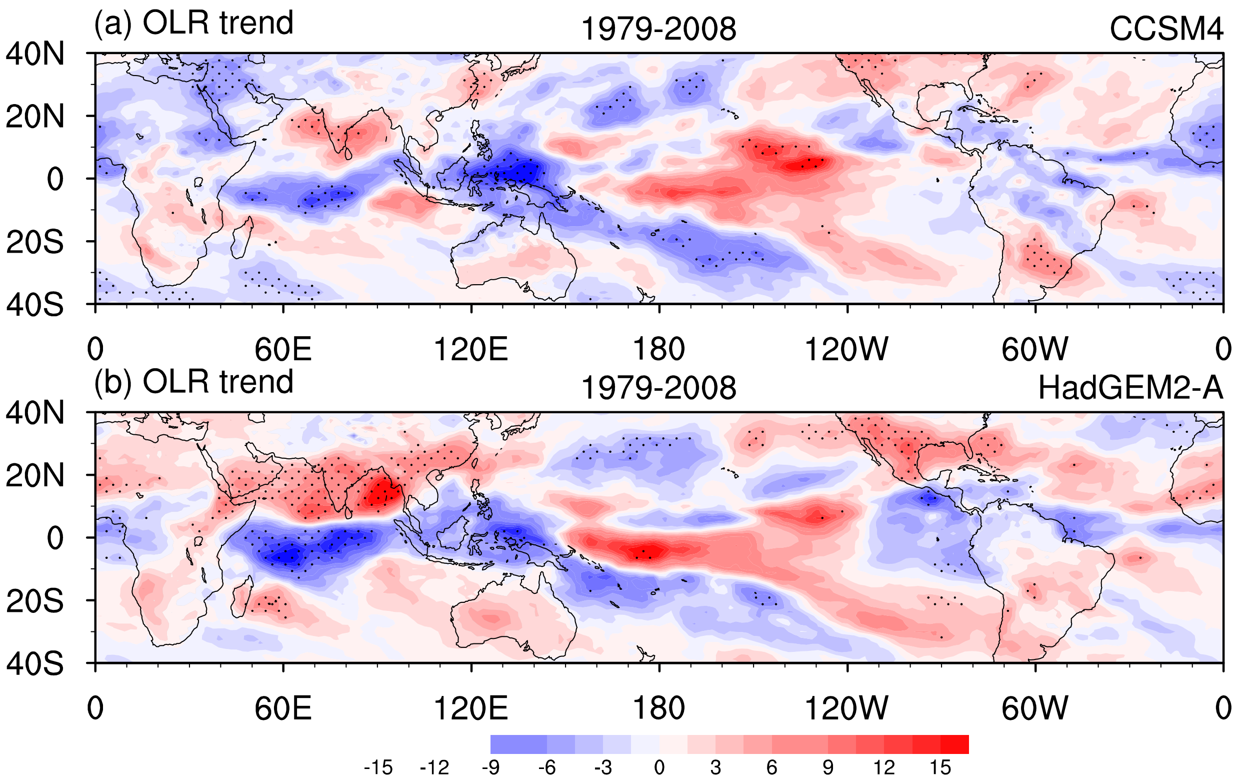

Supplementary Fig. 7 Spatial pattern of changes in annual-mean outgoing longwave radiation (OLR; unit: W/m^2) from AMIP simulations of (a) CCSM4 and (b) HadGEM2-A during 1979-2008. The stippled areas indicate that the change is significant at the 5% level.

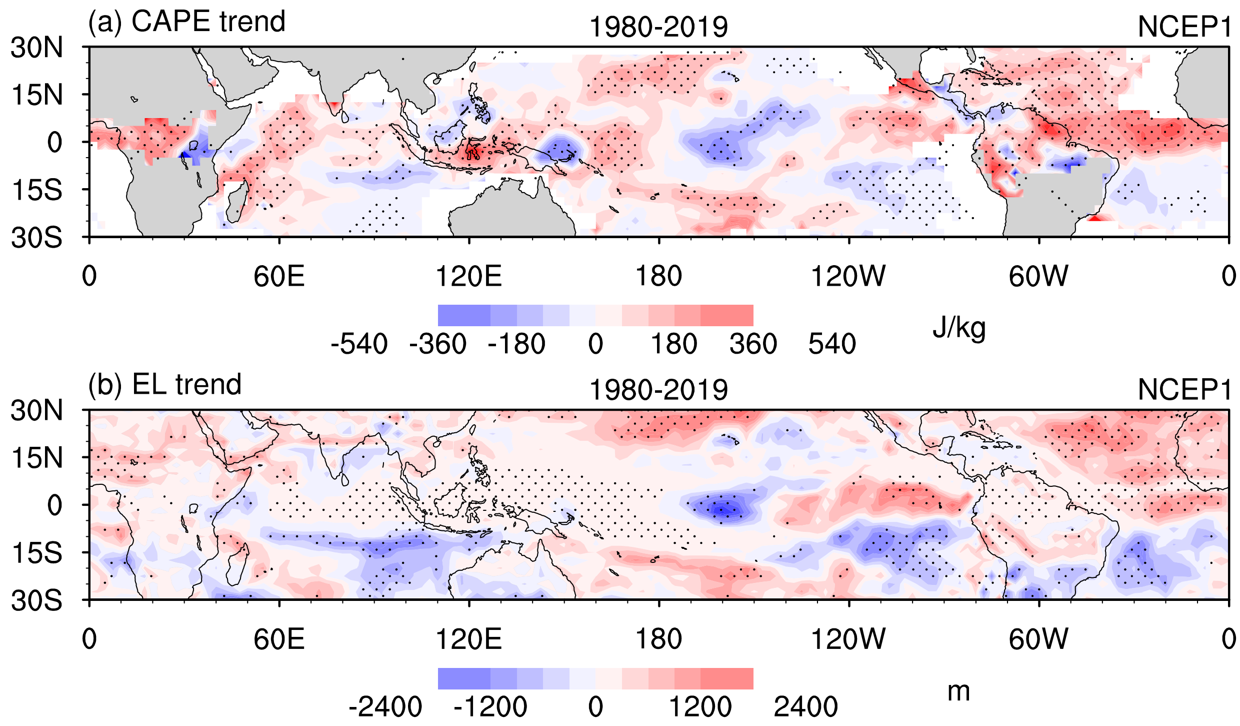

Supplementary Fig. 8 Spatial pattern of changes in annual-mean (a) CAPE (unit: J/kg) and (b) equilibrium level (unit: m) during 1980-2019 from NCEP1. The stippled areas indicate that the change is significant at the 5% level.

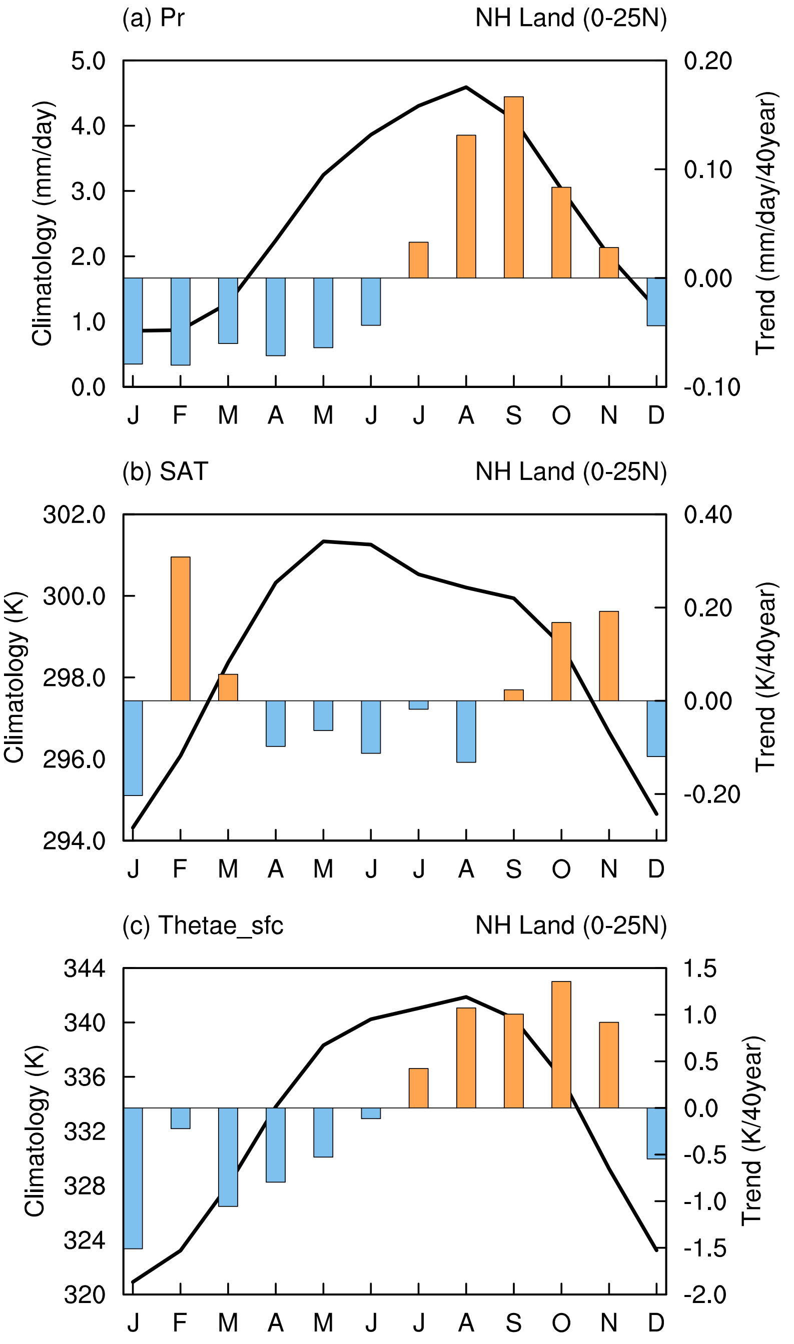

Supplementary Fig. 9 The seasonal cycles (black lines) of (a) precipitation (units: mm $(day)^{-1}$), (b) SAT (units: ^{o}C), (c) Thetae_sfc (units: ^{o}C) over the northern tropical land (0°-25°N) and their trends (vertical bars) during 1980-2019.

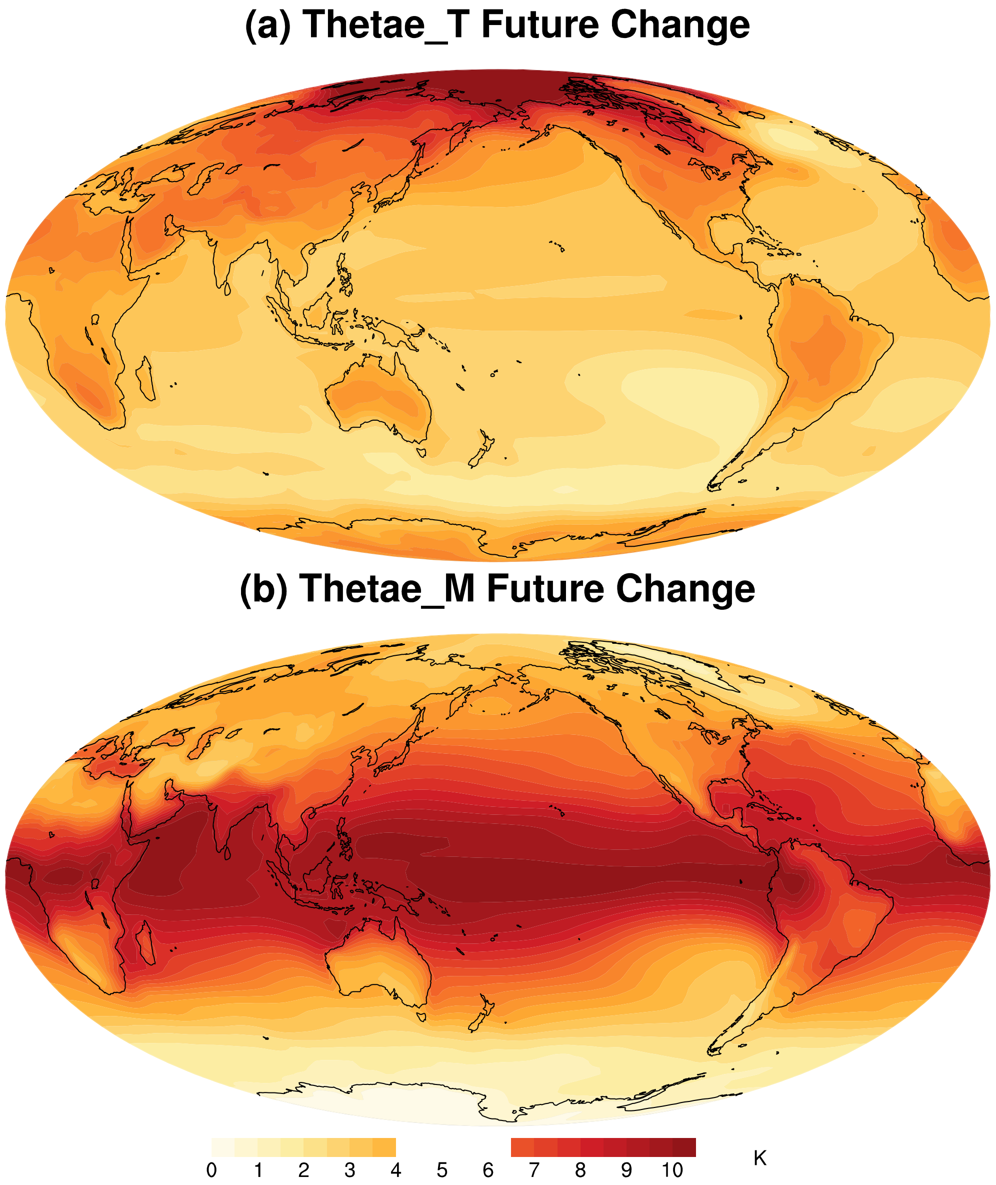

Supplementary Fig. 10 (a) temperature component (θ_e_T; unit: °C) and (b) moisture component (θ_e_M; unit: °C) of surface equivalent potential temperature difference between 2080-2099 mean and 1980-1999 mean under RCP8.5 scenario based on 20 CMIP5 models.

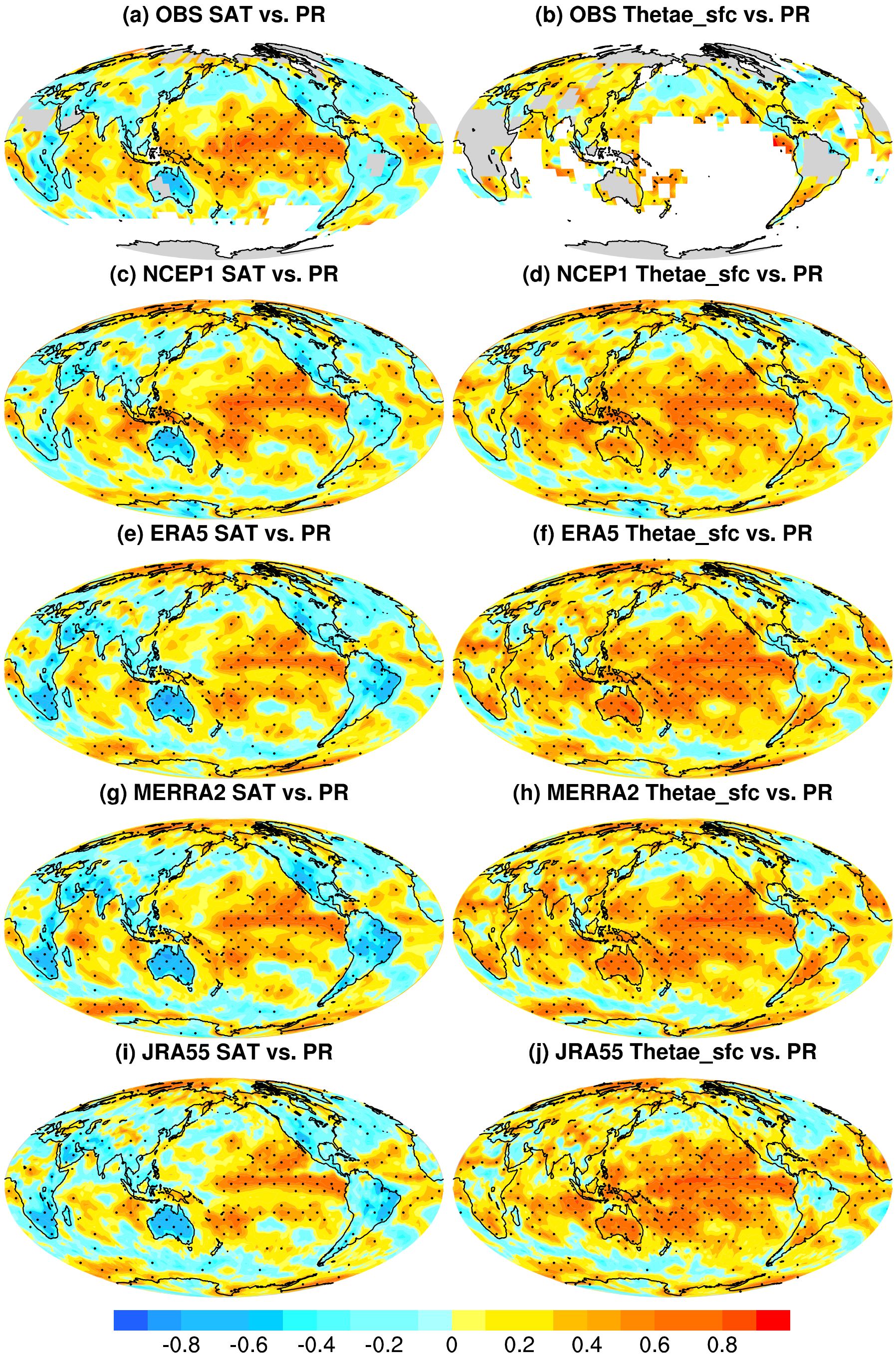

Supplementary Fig. 11 Correlations between (left panel) SAT and precipitation and (right panel) Thetae_sfc and precipitation during 1980-2019 in (a-b) observation, (c-d) NCEP1, (e-f) ERA5, (g-h) MERRA2 and (i-j) JRA55. The correlations are calculated using annual mean data at each grid point.

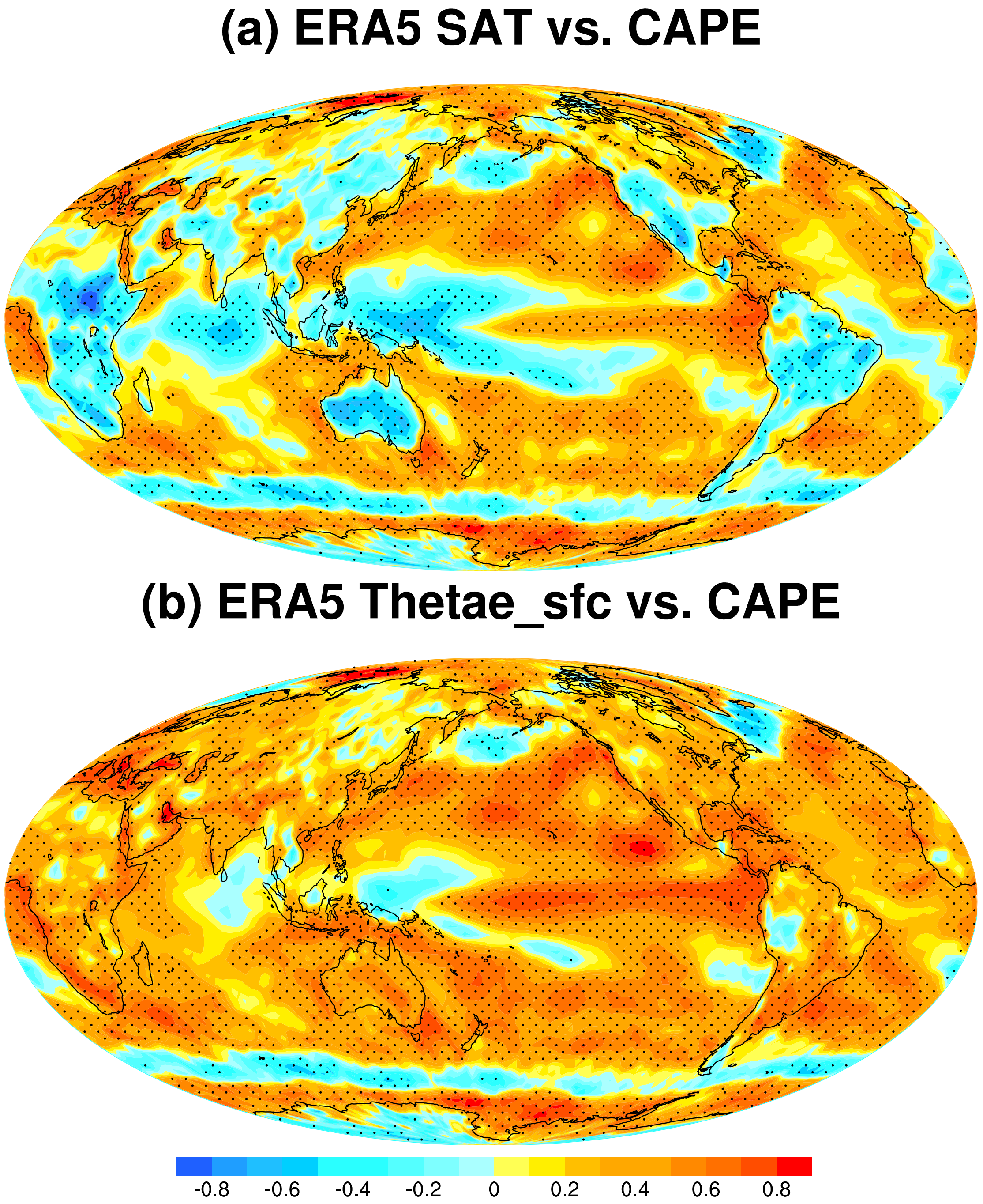

Supplementary Fig. 12 Correlations between (a) SAT and CAPE and (b) Thetae_sfc and CAPE during 1980-2019 in ERA5.

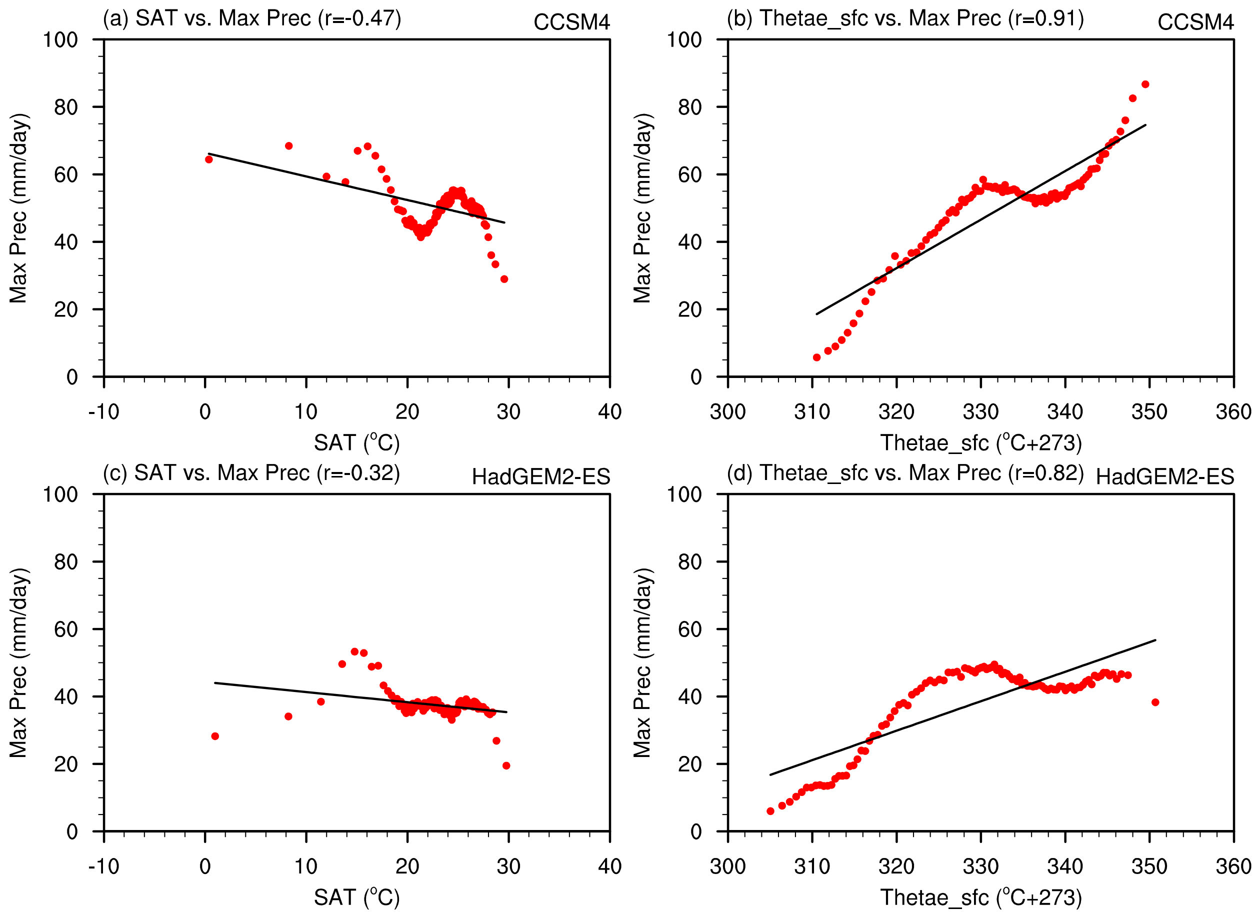

Supplementary Figure 13 Relationship between annual maximum precipitation and annual mean SAT and Thetae_sfc over tropical land (30ºS-30ºN) during 1980-2019 from two CMIP5 models. Top panel is from CCSM4 and the bottom panel is from HadGEM2-ES. Each point represents a bin average of data from all land grid points in the latitude belt. The correlation coefficient is given in the parentheses at the top of each frame.

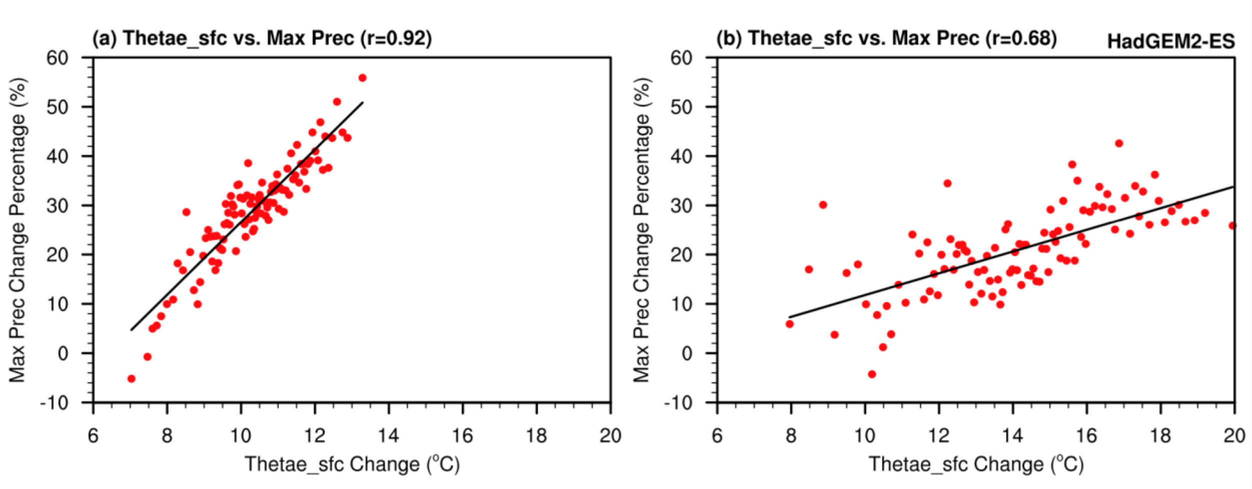

Supplementary Figure 14 Relationships between intensity changes of annual maximum precipitation over tropical land (30ºS-30ºN) relative to current annual maximum precipitation and Thetae_sfc changes at the end of the 21 Century from projections of two CMIP5 models. The maximum precipitation change is the difference between 2080-2099 and 1980-1999. The same model grid points as in Supplementary Figure 13 are used here.

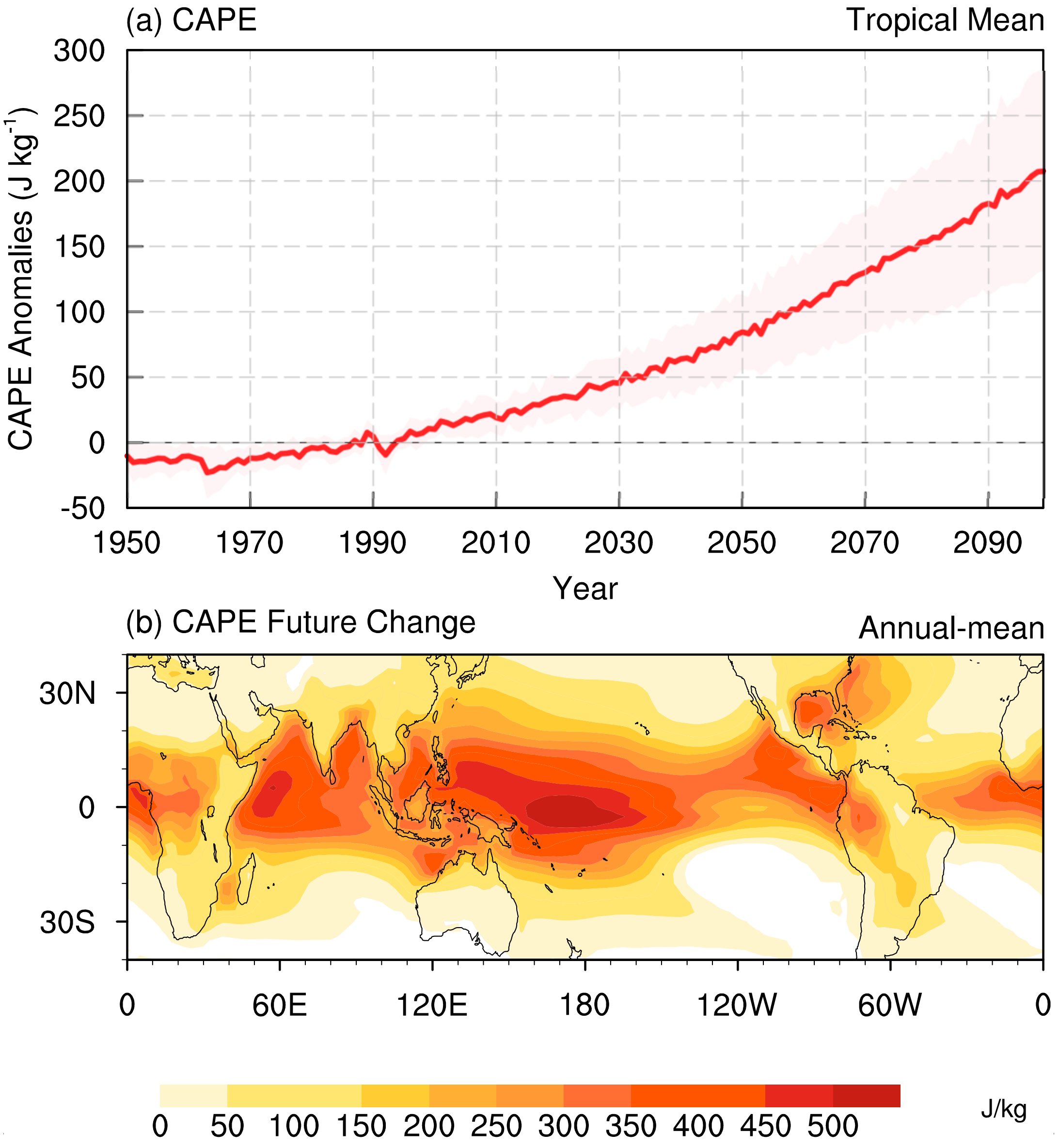

Supplementary Fig. 15 The future changes of CAPE (unit: J kg^{-1}) and its spatial distribution. (a) Annual-mean time series of CAPE averaged in the tropics (30°S-30°N) relative to 1980-1999. (b) The future change pattern of annual-mean CAPE between 2080-2099 and 1980-1999.

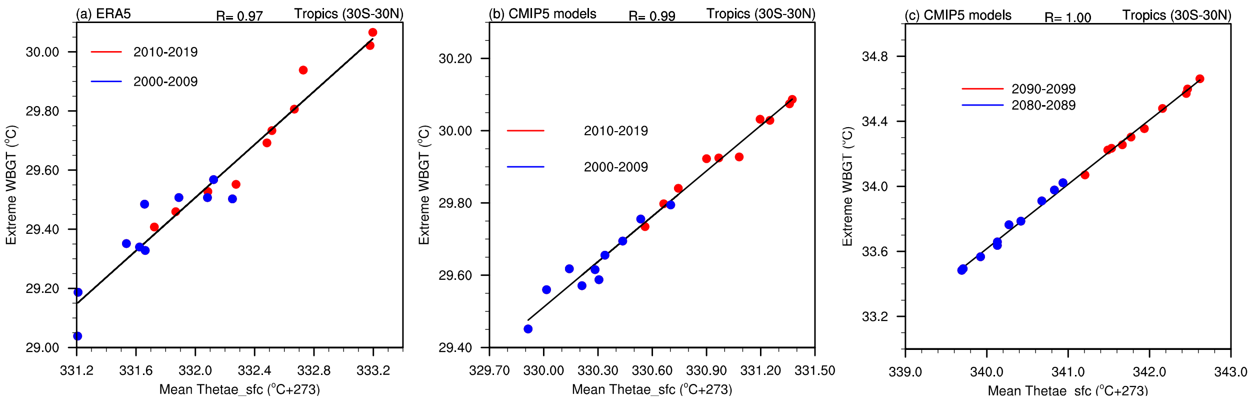

Supplementary Figure 16 The extreme WBGT is highly correlated with mean Thetae_sfc, with the hotter decade having higher extreme WBGTs. a) The extreme WBGT over land is highly correlated to time-mean Thetae_sfc in the tropical land (30S-30N) from the ERA5 Reanalysis data during the last two decades. Blue points are for the decade 2000-2009 and red points are for 2010-2019. Extreme is defined as 5% hottest. b) Same as a) but from CMIP5 models, c) Same as b) but for the two decades from 2080-2099 period.

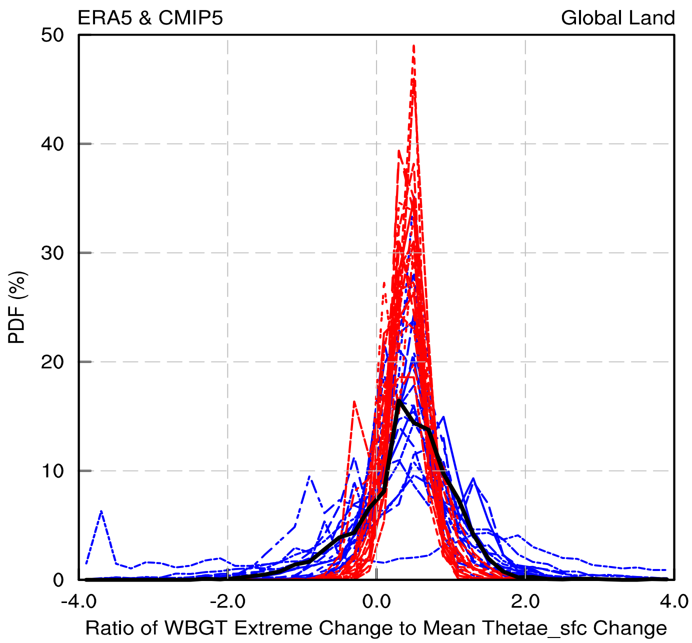

Supplementary Fig. 17 The probability distribution function (%) of the ratio of WBGT extreme temperature trend to the annual-mean Thetae_sfc trend over the global land during 2000-2019 in the ERA5 (black line), 2000-2019 in the 19 CMIP5 models (blue lines) and 2080-2099 in the 19 CMIP5 models (red lines).

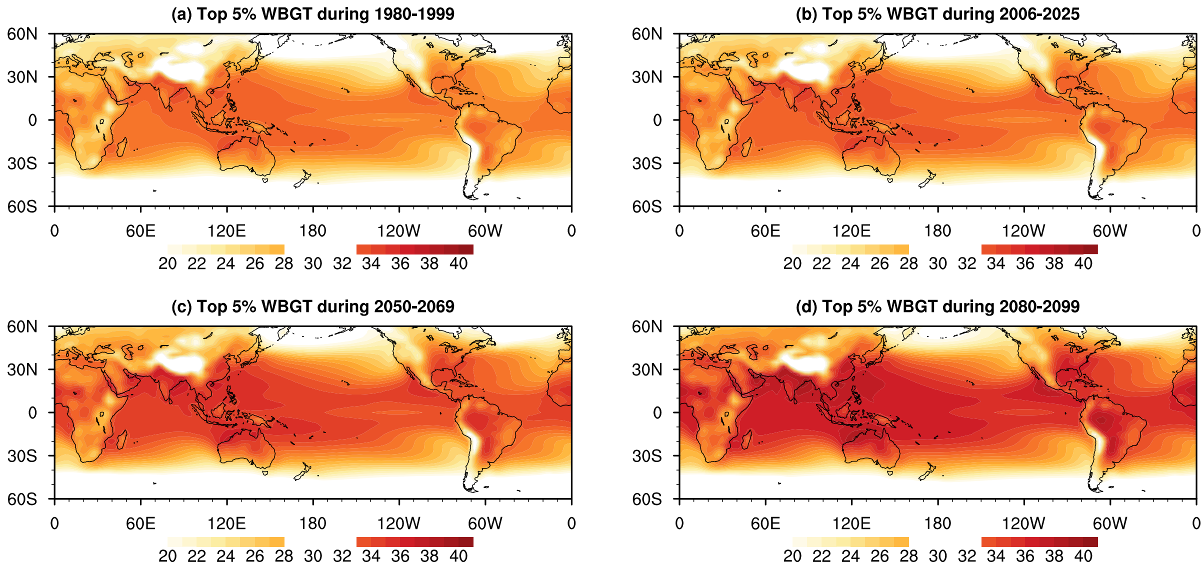

Supplementary Fig. 18 The extreme surface WBGT (unit: °C) defined as the top 5% percentile during (top row) 1980-1999, (second row) 2006-2025, (third row) 2050-2069 and (bottom row) 2080-2099 during boreal summer (June-August) from 19 CMIP5 models.

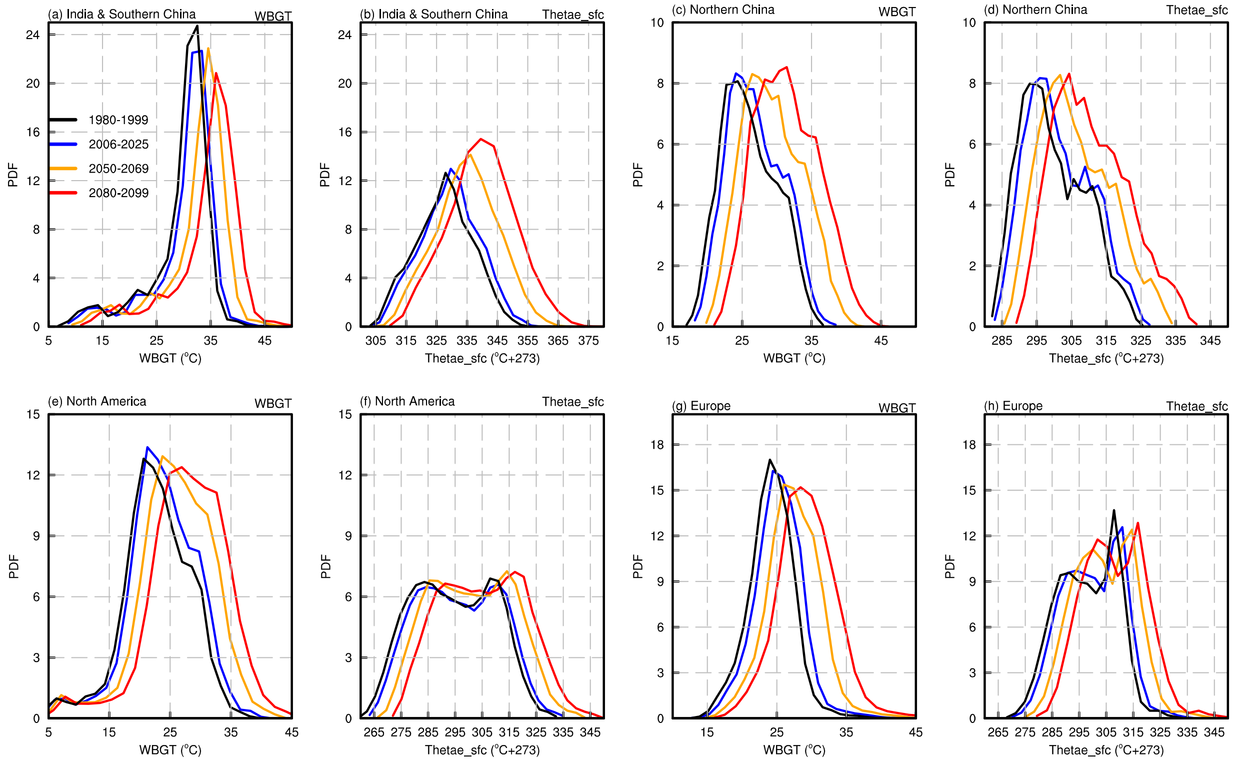

Supplementary Fig. 19 The PDF of annual-mean surface WBGT (unit: °C) and surface equivalent potential temperature (unit: °C+273) over India and Southern China (10°-30°N,60°-125°E), Northern China (30°-50°N,105°-125°E), North America (30°-65°N,240°-320°E) and Europe (30°-65°N,0°-60°E) from 19 CMIP5 models. The black, blue, orange, and red lines represent 1980-1999, 2006-2025, 2050-2069 and 2080-2099, respectively.
